# On Phlebitis of the Great Venous Trunks of the Neck Subsequent to Labor*Read at a meeting of the Association of the King and Queen’s College of Physicians in Ireland, May 7, 1856.

**Published:** 1856-10

**Authors:** Alfred H. M’Clintock

**Affiliations:** Licentiate of the King and Queen’s College of Physicians in Ireland; Master of the Lying-in-Hospital, Dublin, &c.


### RECORD OF MEDICAL SCIENCE.

*On Phlebitis of the Great Venous Trunks of the Neck subsequent to Labor.** By ALFRED H. M'CLINTOCK, M. D., M. R. I. A., F. R. C. S. I., Licentiate of the King and Queen's College of Physicians in Ireland; Master of the Lying-in-Hospital, Dublin, &c.

The study of venous inflammation has, of late years, deeply engaged the attention of pathologists; and their discoveries, in this field of inquiry, have already thrown considerable light upon the nature and connexion of many obscure diseases. By these investigations both the practical surgeon and physician have been largely benefited; whilst the obstetrician has derived from them the greatest possible assistance, in unfolding the mysterious intricacies of puerperal fever, and explaining many of the otherwise inexplicable circumstances and features of this complex malady.

The two nearly related subjects—phlebitis and pyemia—both still require close investigation, and offer for solution many important problems. Every new fact, therefore, in their history—every novel aspect under which either disease is seen—should be placed upon record, and so rendered available to aid in maturing our knowledge, and forming a basis for scientific generalization. Accordingly I venture to bring forward the following clinical history, as it presents some features of extreme rarity and deep practical interest.

S. D., aged 22, was confined of her first child, a boy, on March 8th, after a labor of twenty-eight hours' duration. The consumption of time chiefly took place in the first stage, and in consequence of rigidity of the os uteri. For several days previously to the setting in of true labor, she suffered much from spurious pains; and very considerable hemorrhage occurred immediately after the expulsion of the after-birth. From the time of delivery her pulse was remarked to be quick, above 100; and this rate of frequency did not subsequently diminish. During the ensuing week she remained in a very unsatisfactory state. She had no rigor, nor anything like a distinct accession of fever or inflammation; but the pulse was constantly 110, or upwards; the belly was tumid; the uterus large, and tender on deep pressure; and she was troubled with painful tenesmus, and frequent dysenteric stools. Nevertheless, she was cheerful, and made no complaint of uneasiness in any particular situation. On her ninth day there was an apparent improvement in every respect, the pulse falling to 96, the belly becoming soft, and bowels being moved only five or six times in the twenty-four hours. On her tenth day she drew our attention to a fulness and tenderness immediately above the inner end of the right clavicle. There was a slight tumefaction in this situation, such as might be caused by a

* Read at a meeting of the Association of the King and Queen's College of Physicians in Ireland, May 7, 1856.

simple glandular enlargement. About this time she began to complain of a short, irritating, dry cough, which used to annoy her in the evening, and for which no satisfactory cause could be discovered. A few days later a similar swelling appeared in the corresponding situation on the left side of the neck. She did not seem to experience any serious uneasiness from these tumors, and beyond fomenting with warm water, and painting with tincture of iodine, no decisive treatment was employed. Some days later—namely, on the 22d March—her fifteenth day—I detected the existence of considerable œdema at the root of the neck, and across the upper and anterior part of the chest, but more so at the left side. The diarrhœa still continued, though with greatly diminished severity; her pulse was usually about 108; and the abdomen was flaccid, and entirely free from pain or fulness.

On the 26th March the œdematous swelling of her neck was decidedly less, though her face seemed somewhat puffy, and the tenderness still remained. As she was now eighteen days confined, and seemed better, we yielded to her own urgent request for leave to be dressed, and to lie on the outside of the bed. Before she was dressed, however, she got an indistinct rigor, and had to be put back into bed.

From this time forward her symptoms underwent a striking change; and she progressively became worse and worse. There was a marked exacerbation of all the febrile symptoms; with hurried respiration, and occasional vomiting. In addition to these it was noticed, that the superficial veins, beneath the clavicles, and on the forepart of the chest, had manifestly become varicose.

From a careful review of her history, and an attentive consideration of her present symptoms, there seemed little room to doubt the existence of inflammation of some of the deep veins of the neck; and though not aware that such a diagnosis had ever been made, still we conceived that no other lesion could satisfactorily explain the peculiar features of her case.

On March 28th she is reported to have slept tolerably well; pulse 120; a deep flush on each cheek; the rest of the face is puffy, and of a chlorotic hue; the belly is soft and relaxed, and everywhere free from tenderness; the network of enlarged veins is still more apparent than heretofore, and includes some branches on the upper arm. The tongue is dry, and has a brown streak down the centre. Her breathing somewhat oppressed; and she continues to have towards evening a short teasing cough; this seems to be her chief source of discomfort; for, unless she moves her head, she experiences no pain in the neck. A slight rigor occurred to-day; in the evening it was remarked that her hearing was somewhat impaired. A rough bruit was detected with the first sound of the heart.

Her condition on the next day was, in every respect, more alarming; the pulse 128, and weak; tongue dry, brown, and crusted; slight subsultus of hands; frequent sickness of stomach; face much swollen; eyes prominent and pupils dilated; deafness increased towards evening; she complained of indistinctness of vision. Her mind continues clear and undisturbed.

On the 30th it was plain that her dissolution could not be far off. The respirations were frequent and labored; the pulse scarcely countable, and very weak, though the heart was beating strongly; a thick brown crust on the tongue; pupils very much dilated. She was drowsy, and towards evening lapsed into a comatose state, which ended in death at 9 P.M.

*Autopsy*, twelve hours after death.—The enlarged veins in the neck and chest still very distinct, though changed in color. The abdomen, when laid open, presented no morbid appearance; the liver was healthy. The *uterus* was rather large for this period—twenty-two days—after delivery; its structure was remarkably soft and friable. Behind this organ, and deep in the pelvis, existed a small abscess. The mucous membrane of the large intestines seemed thickened, and was of a very dark color, apparently from intense congestion. The *kidneys* were in an advanced stage of fatty degeneration. On dissecting back the integuments of the neck, and exposing the great venous trunks in this region, it was at once apparent that phlebitis, in its most marked form, had existed here. The deep jugulars, both subclavians, the upper part of each axillary, the right vena innominata, and superior portion of left, were the vessels engaged. In calibre they seemed enlarged, their coats were thickened, and internally they contained firm plugs of coagula and lymph, the latter being the more external, and adhering very closely to the walls of the vessel. These formations extended down to near the superior cava, the lining membrane of which was redder than natural. The pulmonary valves were intensely red, as was the interior of the pulmonary artery,—contrasting strongly with the aorta, which presented its natural color. In the right auricular appendix was a small incipient abscess. The tricuspid and mitral valves were intensely injected, so as to present a bright scarlet color. The aortic valves contained some calcareous matter, but otherwise presented no abnormal appearance. The lining membrane of the heart itself was pale, and showed no traces of inflammation.*

Such is a very brief history of the symptoms and morbid appearances which this case presented. It will be remarked that, at an early period after parturition, the patient became affected with symptoms of low puerperal fever, the gastro-intestinal mucous membrane being the part on which the action of the poison seemed to be chiefly expended, though it is possible this diarrhœa may have had some connexion with the renal disease. A variety of circumstances concurred in this case to favor the development of puerperal fever. The kidneys were far advanced in fatty degeneration; she had spurious pains for a considerable time before the setting-in of true parturient action; her labor was tedious (twenty-seven hours), and was followed by severe hemorrhage. Another circumstance there was in her case, yet more influential than

* The preparation showing these morbid alterations was exhibited to the meeting, and is now in the Museum of the Lying-in-Hospital.

all these together, in predisposing to the invasion of puerperal fever,—she had been seduced, and was laboring under intense mental depression, from the conjoint influence of bitter disappointment, and the cheerless prospect of a life of irretrievable disgrace and shame.

The attack of puerperal fever seemed partially to yield to treatment; there was some mitigation in the local and general symptoms. In the middle of the second week, however, the first indication of phlebitis of the neck showed itself. Now, in most cases of crural phlebitis, we find the course of events to be much the same as that just described. Thus, of sixty cases of puerperal phlegmasia dolens, collected by Dr. Mackenzie, the attack followed upon some form of puerperal fever, in thirty-three instances, and even this proportion I believe to be much under the mark.

The local effects of phlebitis were present, for some days, without being attended with any remarkable constitutional symptoms; and I freely confess that no suspicion crossed my mind, at this period, of the real nature of this swelling in the neck. To this localized tumefaction œdema succeeded; then came a rigor, and after it a sudden explosion of alarming symptoms, which too plainly revealed that the very fountain of life itself was poisoned. At a later period the dilated condition of the superficial veins on the front of the chest attracted our attention, and first suggested the possible existence of phlebitis of the deep veins of the neck. But little reflection was required for this conjecture to settle down into absolute conviction; in fact, for the establishment of this diagnosis, no symptom was now wanting: there were local swelling and tenderness, œdema, unequivocal signs of venous obstruction, and the constitutional disturbance ordinarily attendant upon phlebitis. Mark, also, the sequence in which the symptoms appeared:—First, puerperal fever, and apparently partial recovery; next, localized pain and tumefaction; then œdema; and, some days later, a rigor, with increased constitutional disturbance; and, lastly, varicose enlargement of the superficial veins.

The condition in which the deep jugular veins were found, after death, plainly showed that they had become wholly impervious, so that the return of blood from the head must have been entirely effected through the vertebral and superficial jugular veins. That great obstruction existed in the venous circulation, was manifest during the last few days of her life, and caused the aspect and expression of her face to bear a very close resemblance to those cases in which heart disease, and enlargement of the thyroid-gland, co-exist. I regret exceedingly that an examination of the head could not be obtained.

The subclavian vein on each side was more or less obstructed by firm coagula and lymph; yet there was no œdema of either hand or either arm. It is, perhaps, impossible to say, with certainty, in what particular spot the phlebitis began, or in what direction it spread; that is, whether in a direction towards or from the heart. My own opinion is, that it extended along the veins *contrary* to the course of the circulation, as is generally observed in crural phlebitis. This opinion is founded on observation of the situation of the tumor during life, and of the morbid appearances in the veins,—the apparently more recent inflammatory deposits being at the remote point from the heart.

The existence of fatty degeneration of the kidneys is a feature in this case that should not be overlooked, particularly at the present time, when so much attention is being directed to the influence which this organic lesion exercises over the progress and results of intercurrent diseases. The urine was not at any time tested, as, owing to the constant presence of diarrhœa, it was not procured free from the admixture of fæcal matter.

I believe it will not be an exaggeration to assert, that the case just detailed, in so far as relates to the phlebitis of the venæ innominata, is almost unique in medical literature. I know of but one similar instance; it was exhibited at the Pathological Society, in November, 1851, by Dr. Mayne, and the account of it is published in the thirteenth volume of the Dublin Quarterly Journal, N. S.

From its interest and great practical importance, I feel that no apology is necessary for quoting Dr. Mayne's account of this case, as submitted to the Pathological Society :—" The case was that of a man aged 29, who had been in hospital in February last, laboring under symptoms of incipient phthisis. After a few weeks he left the hospital, and returned to his business; he was very soon, however, seized with a severe bowel complaint, supposed by himself to be dysentery, and which persisted up to last October, when he again placed himself under Dr. Mayne's care, in consequence of the sudden supervention of pain and swelling in the *right* upper extremity; the pain, commencing behind the clavicle and in the corresponding shoulder, was soon followed by œdematous tumefaction of the entire limb, which was hot, painful to the touch, and pitted imperfectly upon pressure. The deep-seated veins could be traced, hard and cord-like, under the integuments, and the superficial veins about the shoulder, the axilla, and the arm, were largely dilated, and by their blue color and varicose appearance at once attracted the attention of the observer. For the few days during which the man survived, the bowel irritation seemed to supersede the chest symptoms. The stools, which were as many as twelve to twenty in the twenty-four hours, were of an ochery character, and insupportably offensive.

" At the post-mortem examination both lungs were found filled with tubercles, but there was no cavity of any extent in either. All the veins of the right upper extremity were filled with firm coagula, which adhered to the lining membrane, and retained a considerable portion of the coloring matter of the blood. These coagula, when removed from the veins, resembled portions of coral. The coats of the veins were thickened and opaque. These diseased appearances extended as far as the junction of the two venæ innominatæ; the right vena innominata was quite impervious, while the left, the superior cava, and the azygos, were free from any trace of inflammation. The examination of the small intestines was accidentally omitted, but the large intestines exhibited the appearances usually observed in the advanced stage of chronic dysentery; the mucous membrane was covered with ulcers, many of which in the rectum and sigmoid flexure of the colon were circular, with depressed centres and indurated margins."

Whilst this case resembles, in some respects, the one I have above related, still, between the two there are several points of difference, but which it is needless here to enter upon.

In neither of them was the phlebitis a primary, idiopathic disease. In Dr. Mayne's case it was preceded by phthisis and chronic dysentery; in my case, by puerperal fever and kidney disease.

Respecting the etiology of phlebitis our knowledge is as yet very imperfect. That it may arise from the wound or injury of a vein, is clearly established; though atmospheric condition, and the health of the individual at the time, exercise a powerful influence in modifying the results of this injury. Secondly, phlebitis may result from the immediate contact of purulent or septic matter with the endangium of a vein. That this is not by any means a necessary consequence, however, is abundantly proved by the experiments of Gaspard, Cruveilheir, and Mr. Henry Lee; on the contrary, the blood may be contaminated, and pyemia induced, without any inflammation of the venous canals through which the poison gained an entrance into the circulation. Thirdly, there is good reason to suppose that phlebitis, under certain circumstances, may originate in an extension of inflammation from surrounding tissues. Veins imbedded in bone would appear more disposed than others thus to become implicated in the inflammation of contiguous structures.

Lastly, we sometimes see inflammation affecting venous trunks, independently of any of the preceding causes, and where its production may, in a limited sense, be considered spontaneous; whether or how far it is truly idiopathic, is another and more difficult question to determine. I am strongly inclined to the opinion that phlebitis, thus arising, is not essentially idiopathic; that it is only a consequence, a local manifestation, of toxæmia, or vitiated blood. The circumstances under which this so-called spontaneous form of phlebitis presents itself, lend considerable support to the above view. It is never seen occurring, for instance, in the midst of sound health, but attends or follows upon various diseases whose decided tendency is to alter and deteriorate the blood. In this category we have typhoid, adynamic, and puerperal fevers; phthisical, gouty, and rheumatic states of the constitution; chronic dysentery and chlorosis. Dr. Robert Lee maintains that phlegmasia dolens (or crural phlebitis) is always an extension of inflammation from the venous textures of the uterus. This, be it remembered, is the opinion of one of the highest authorities upon this subject. In it we see a recognition of the fact, of crural phlebitis being *preceded* by a disease eminently favoring the production of blood poisoning. Whether this extension of morbid action does or does not take place, we need not now stop to argue, as the general question is thereby affected in but a small degree.

Dr. Mackenzie's researches on the nature and pathology of phlegmasia dolens have, I think, a close relation to the point now under consideration. He does not deny that phlebitis is always present in the affected limb. This, indeed, is a fact as well established as any other in pathology; but what he contends for is, that this inflammation of the veins is secondary to, or arises out of, a vitiation of the circulating fluid. In confirmation of this he adduces the results of many direct experiments, and cites a large number of cases of phlegmasia dolens, from various authors, all concurring to give strong support to his pathological views.

I am not going to pursue this inquiry further at present, as it would open up too wide a field for research, and one, moreover, which, to a certain degree, has already been explored by Dr. Mackenzie. In the case already related, and which has led to these remarks, there can be no possible doubt that when the phlebitis of the neck appeared, a state of pyemia, or at least of blood-poisoning, had for some days existed. Her symptoms then, and previously, together with the post-mortem appearances, concur in supporting this view.

In Dr. Mayne's patient the health was broken down and seriously impaired by chronic dysentery and phthisis, for a considerable time previously to the occurrence of the phlebitis; so that we see, in both these remarkable instances, causes were in operation quite adequate to produce an altered, vitiated state of the blood.

It has been stated, in the course of the clinical history of this interesting case, that the local effects of phlebitis were present for some days without being attended with any remarkable or peculiar constitutional symptoms. This leads me to observe here, that of *pure phlebitis we have, I believe, in point of fact, no pathognomonic symptoms beyond the local ones; the symptoms considered to be such belonging rather to the state of pyemia.* Hence, in all cases where a vein or veins, beyond the reach of tactile examination, are inflamed, as, for example, in a case of uterine phlebitis, we cannot know of a certainty that the venous tissue is the one particularly engaged, unless the characteristic symptoms of pyemia show themselves. To this conclusion, at least, I am led by my own experience. I may, perhaps, be in error on the point, and therefore would not wish to speak dogmatically. At all events it is just one of those questions which can only be decided by the collective experience of many observers. It, therefore, with the other question—" Does phlebitis ever occur as a purely idiopathis disease?"—I would respecfully throw out, as subjects on which it would be of advantage to elicit the opinions of others.—*Dub. Quarterly Journ.*

